# Compassionate Curiosity: Mitigating the Effects of Bias Through Motivational Interviewing

**DOI:** 10.1089/heq.2024.0054

**Published:** 2024-06-13

**Authors:** Paul J. Hershberger, Dean A. Bricker, Angie Castle, Timothy N. Crawford, Stacy R. Flowers, Alexandria L. Goff, Katharine Conway

**Affiliations:** ^1^ Department of Family Medicine, Wright State University Boonshoft School of Medicine, Dayton, Ohio, USA.; ^2^ Department of Internal Medicine, Wright State University Boonshoft School of Medicine, Dayton, Ohio, USA.; ^3^ Department of Population and Public Health Sciences, Wright State University Boonshoft School of Medicine, Dayton, Ohio, USA.; ^4^ Wright State University Boonshoft School of Medicine, Dayton, Ohio, USA.

**Keywords:** motivational interviewing, implicit bias, patient engagement, chronic disease management, health professions training

## Abstract

There is strong evidence that the implicit biases of health care professionals affect the treatment of patients, and that minority and other marginalized patients are disproportionately harmed. Assumptions made about patient knowledge or lack thereof function as judgments that are prone to bias, which then affect the education and advice imposed upon patients. We review how the motivational interviewing (MI) approach to patient engagement includes components of evidence-based bias-mitigating strategies, such as understanding circumstances from the patient’s point of view, and therefore we propose that the MI approach can reduce the impact of bias in patient care.

“First do no harm.” This phrase, widely associated with the Hippocratic Oath even though it was not in the original document, emphasizes the importance of avoiding deleterious behavior on the part of physicians with their patients.^1^ This includes efforts to minimize the influence of biases in patient care, both explicit biases (those of which one is aware) and implicit biases (those outside one’s awareness). Health care professionals may not want to acknowledge that they have biases toward patients, nor that they are judgmental, but the evidence suggests that they have the same level of implicit bias as the general population.^2,3^ Clinician biases impact diagnoses and treatment decisions, leading to substandard care, especially for ethnic minority, gender or sexual minority, poor, higher weight, mentally ill, and other vulnerable patients.^3,4^ We propose that the use of the motivational interviewing (MI) approach is a means to reduce the impact of bias in patient encounters.^5^ While largely unstudied, we postulate that the MI style can help reduce bias as the approach includes components of evidence-based bias-mitigating strategies.

## Biases and Health Inequities

Chronic illness is the primary health burden in the United States, as 60% of adults have at least one chronic condition and over 40% have more than one.^6^ Racial and ethnic minorities fare the worst.^7,8^ Despite the United States spending substantially more on health care than any other developed country, with 90% of these expenditures going for chronic illness and mental health, the United States is approximately two standard deviations below the mean in preventing chronic disease morbidity.^9^ Engagement of the patient in taking responsibility for health-related behaviors is crucial, as the management of health is primarily the responsibility of the patient.^10,11^ Health professionals commonly want to help their patients and “fix” their problems, making it seem natural to tell patients what to do. Accordingly, a common approach taken by clinicians to address their patients’ behavior is to reflexively educate and advise them, recommending steps that they should take to improve their health.^12^ Yet the “educate and advise” approach to patient behavior appears to be ineffective, perhaps in part because self-rated health in the United States is quite high,^9^ meaning that many adults may not perceive any need to change health-related behavior.

Furthermore, educating and advising typically involves making assumptions about patient knowledge or lack thereof, because what the patient knows may not be elicited from the clinician. These judgments about what patients need to know or do are prone to the impact of implicit biases. To the extent that patients experience this approach as judgmental, they may tend to be less forthcoming with clinicians, potentially withholding important health-related information.^13^ Time pressures are ubiquitous in health care, and there is evidence that patients are finding that the quality of physician communication is declining, including less listening.^14^ Patients may be doing less talking, and the majority of adults do acknowledge that they have not been forthright with their clinician. The most prevalent reason for such nondisclosure was “I didn’t want to be judged or get a lecture about my behavior.”^13^ Furthermore, communication quality is typically found to be poorer with patients of color, particularly with respect to not communicating incidental findings.^15^

Alternatives to the “educate and advise” approach to patient behavior can have a positive effect on patient outcomes. Collaborative treatment plans that incorporate patient values, goals, and priorities are associated with higher levels of patient activation, followed by better adherence and improved health outcomes.^16^

## Motivational Interviewing

MI is an evidence-based, brief interventional approach to patient engagement, found to be highly effective in increasing patient activation.^5,17^ The MI approach involves patient-focused conversation that reinforces the patient’s motivation to make positive health behavior changes. The patient’s normal and natural ambivalences to change are explored, and through a guiding style the clinician supports the patient in developing a need for change.^18^ The MI approach emphasizes being nonjudgmental—compassionately curious—to elicit the patient’s perspective on change and take responsibility for their choices. Compassion requires an awareness of another’s distress together with a desire to help alleviate it. Curiosity necessitates an inquisitive interest in the components of the other’s distress. Listening to the patient is a crucial skill, especially when attention is given to what the patient values and where their current behavior is discordant with what they care about. Such an approach to patient interaction on the part of clinicians is crucial to the promotion of health equity.

The World Health Organization (WHO) considers health to be a fundamental human right and asserts that “health equity is achieved when everyone can attain their full potential for health and well-being.”^19^ Furthermore, in order to improve health equity, the WHO proposes “evidence-informed action… to ensure high-quality and effective services are available…”^19^ A high-quality and equitable health care environment in which all persons can attain optimal health and wellness requires clinician–patient interactions in which bias and judgment are minimized, and in which patients experience the psychological safety necessary for them to be honest about their health-related behavior, beliefs, challenges, concerns, and priorities.^20^

The MI approach is evidence-based for chronic disease management, and the core communication components of the approach can help mitigate the impact of biases. These include developing a trusting relationship with the patient, individuating (i.e., getting to know the patient’s personal values, goals, and challenges), and understanding the patient’s perspective. Acknowledging the presence of bias, and practicing skills such as individuation and perspective-taking, can lessen the impact of bias,^21–23^ although these processes do require careful listening to patients. The kind of listening involved in the MI approach aligns with several key bias-mitigating strategies reported in the behavioral science literature: individuating, perspective-taking, and increasing opportunities for contact.^22,24^ Individuating involves seeking to understand each person as an individual, not simply as a member of a stereotyped group, and paying attention to characteristics about them rather than discriminatory generalizations of their group. This may be particularly important when there is gender, racial, or age discordance between the clinician and patient. Perspective-taking involves compassion and envisioning from the patient’s point of view. Increasing contact includes seeking opportunities to know and interact with people who are different from oneself. The MI approach can help impede assumptions that may be based on stereotypes associated with patient characteristics, such as race, gender, sexual orientation, social class, or diagnoses. When a person from a marginalized group is known and understood as an individual, there are fewer knowledge gaps to fill using stereotypical characteristics for the group(s) of which that person is a member. Importantly, recent research indicates that patient activation lessens the impact of provider bias, and this is likely due to the clinician knowing more about the patient as an individual.^25^

In practicing the MI approach, a clinician is developing habits that reinforce bias-mitigating behavior change. Eliciting the patient’s perspective on treatment options and/or potentially beneficial lifestyle changes can be a way to better understand the social, economic, and environmental influences in the patient’s life. For example, inviting the patient’s thoughts about adding a second medication for hypertension may lead to the patient expressing concern about cost, transportation barriers to get to the pharmacy, or fear of that particular medication because of a family member having troublesome side effects to that medication.

## Conclusion

In health professions training and continuing education programs, teaching learners how to interview and engage with patients is commonly understood as a separate part of the curriculum than are efforts to minimize behavioral manifestations of implicit biases. Our perspective is that training health professionals in an effective patient engagement approach, such as MI, is itself a fundamental way to develop skills that simultaneously help mitigate the effects of implicit bias and contribute to better health outcomes for marginalized patients. The compassionate curiosity that is fundamental to MI similarly contributes to the individuation processes that are critical for health equity.

## Abbreviations Used

MI: motivational interviewing
WHO: World Health Organization
